# Dysfunction of outer segment guanylate cyclase caused by retinal disease related mutations

**DOI:** 10.3389/fnmol.2014.00004

**Published:** 2014-02-26

**Authors:** Patrick Zägel, Karl-Wilhelm Koch

**Affiliations:** ^1^Biochemistry Group, Department of Neurosciences, Carl von Ossietzky University OldenburgOldenburg, Germany; ^2^Research Center Neurosensory Science, Carl von Ossietzky University OldenburgOldenburg, Germany

**Keywords:** photoreceptor guanylate cyclase, GCAP, retinal dystrophy, neuronal calcium sensor, phototransduction

## Abstract

Membrane bound guanylate cyclases are expressed in rod and cone cells of the vertebrate retina and mutations in several domains of rod outer segment guanylate cyclase 1 (ROS-GC1 encoded by the gene *GUCY2D*) correlate with different forms of retinal degenerations. In the present work we investigated the biochemical consequences of three point mutations, one is located in position P575L in the juxtamembrane domain close to the kinase homology domain and two are located in the cyclase catalytic domain at H1019P and P1069R. These mutations correlate with various retinal diseases like autosomal dominant progressive cone degeneration, e.g., Leber Congenital Amaurosis and a juvenile form of retinitis pigmentosa. Wildtype and mutant forms of ROS-GC1 were heterologously expressed in HEK cells, their cellular distribution was investigated and activity profiles in the presence and absence of guanylate cyclase-activating proteins were measured. The mutant P575L was active under all tested conditions, but it displayed a twofold shift in the Ca^2^^+^-sensitivity, whereas the mutant P1069R remained inactive despite normal expression levels. The mutation H1019P caused the cyclase to become more labile. The different biochemical consequences of these mutations seem to reflect the different clinical symptoms. The mutation P575L induces a dysregulation of the Ca^2^^+^-sensitive cyclase activation profile causing a slow progression of the disease by the distortion of the Ca^2^^+^-cGMP homeostasis. In contrast, a strong reduction in cGMP synthesis due to an inactive or structurally unstable ROS-GC1 would trigger more severe forms of retinal diseases.

## INTRODUCTION

Inherited retinal diseases are caused by a heterogeneous group of mutations in retinal genes (Perrault et al., 2000; den Hollander et al., 2008; Athanasiou et al., 2013). Among them are a large number of genes coding for proteins involved in phototransduction processes in the outer segments of rods and cones. Genetic heterogeneity is paralleled by diverse phenotypical and clinical characteristics of retinal degenerations, which are classified in disorders like cone-rod dystrophies, retinitis pigmentosa, and leber congenital amaurosis (LCA). These disease forms lead to a loss of visual function, but they differ in the progression of specific visual impairments.

Phototransduction in rods and cones is centered on the light-triggered hydrolysis of the second messenger cyclic GMP, its powerful re-synthesis after illumination and its role as a ligand targeting a cyclic nucleotide-gated channel in the plasma membrane (Pugh and Lamb, 2000; Kaupp and Seifert, 2002; Luo et al., 2008). Phototransduction is further under control of several negative feedback loops that are important for recovery of the photoresponse from illumination and for adjusting the cell performance to ambient background light (Fain et al., 2001). One crucial molecular factor involved in the regulation of phototransduction is Ca^2^^+^, which binds to and dissociates from Ca^2^^+^-sensor proteins under oscillating changes in cytoplasmic Ca^2^^+^-concentration [Ca^2^^+^]. A multi-protein complex operating at the interface between changing concentrations of cGMP and [Ca^2^^+^] consists of a sensory type membrane-bound guanylate cyclase that is regulated by guanylate cyclase-activating proteins (GCAPs; Stephen et al., 2008; Dizhoor et al., 2010; Koch et al., 2010; Koch and Dell’Orco, 2013). So far, mutations in patients suffering from retinal diseases are found in the gene *GUCA1A* encoding the Ca^2^^+^-sensor GCAP1 and *GUCY2D* coding for the outer segment guanylate cyclase type 1 (ROS-GC1) (Kitiratschky et al., 2008; Behnen et al., 2010; Hunt et al., 2010). Previous research was focused on elucidating the molecular causes and cellular consequences of point mutations in ROS-GC1 and GCAP1 and in a further step to test therapeutic strategies involving RNA interference techniques and transgenic mouse lines (Buch et al., 2011; Jiang et al., 2011; Boye et al., 2013).

**FIGURE 1:**
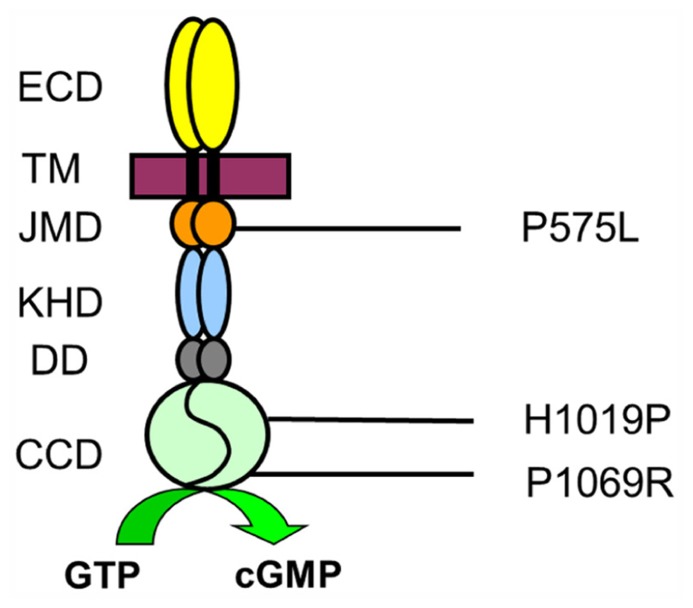
**Amino acid positions of mutations in ROS-GC1**. Topology of ROS-GC1 domains is indicated as extracellular domain (ECD) corresponding to the intradiscal region when cyclases are expressed in rod outer segments, transmembrane domain (TM), JMD, KHD, dimerization domain, and cyclase catalytic domain (CCD). Numbers correspond to the unprocessed human sequence.

However, disease causing mutations can also point to critical amino acid positions that determine structure-function relationships in natively folded protein structures and therefore might help to gain mechanistic insights of protein function and regulation (Duda et al., 1999a, 2000; Ramamurthy et al., 2001). For example, a biochemical study on mutations in the so-called dimerization domain of ROS-GC1 correlating with cone-rod dystrophy recently revealed that this dimerization or linker domain operates as a Ca^2^^+^-sensitive control switch module (Zägel et al., 2013). In the present work we tested the biochemical consequences of three point mutations (**Figure 1**). The mutation P575L was found in a family, where clinically affected members suffer from progressive cone degeneration (Small et al., 2008). The amino acid substitution is located in the juxtamembrane domain (JMD), which is a specific region of sensory GCs upstream of the kinase homology domain (KHD). H1019P is located in the cyclase catalytic domain (CCD) and patients with this missense mutation show the typical clinical symptoms of LCA type 1 suffering from severe visual impairment at birth (Perrault et al., 2000). Finally, P1069R is also within the CCD and a patient carrying this mutation was diagnosed with juvenile retinitis pigmentosa (Booji et al., 2005).

So far, none of these mutations have been investigated on a molecular level. Two of the main questions are for example what the consequences on guanylate cyclase activity are and which Ca^2^^+^-dependent activation profiles can be observed in the presence of GCAPs. These topics are addressed in the present contribution.

## MATERIALS AND METHODS

### CLONING AND SITE-DIRECTED MUTAGENESIS OF HUMAN ROS-GC1 CONSTRUCTS

The cDNA of human ROS-GC1 was cloned into the vector pIRES2-eGFP (Clonetech) exactly as described previously (Zägel et al., 2013). DNA was prepared by using the DNA purification kit of Promega according to manufacturer’s instruction. The mutant P575L was produced by using the following primers for PCR amplification: 5′-GCTATCCGCCTAGCAACCAAGACG-3′ (forward) and 5′-CGTCTTGGTTGCTAGGCGGATAGC-3′ (reverse). Site-directed mutagenesis yielding the mutants H1019P and P1069R was achieved by performing two PCRs each using the ROS-GC1-WT gene in the pIRES2-eGFP vector as template. Primers for the PCR were for creating H1019P: 5′-CCTTACCGCATCCCCGTGAACTTGAG-3′ and 5′-TCAAGAGAACTGGCCCGGCCGC-3′ (forward and reverse primer, first PCR) and 5′-AAAGCTAGCCACCATGACCGCC-TGCGCCCGCC-3′ and 5′-CTCAAGTTCACGGGGATGCGG-TAAGG-3′ (forward and reverse primer, second PCR). P1069R was obtained with 5′-CCCATCCCCAAACGGCCTGACCTG-3′ and 5′-TCAAGAGAACTGGCCCGGCCGC-3′ (forward and reverse primer in the first PCR) and 5′-AAAGCTAGCCACCATGACCGCCTGCGCCCGCC-3′ and 5′-CAGGTCAGGCCGTTTGGG-GATGGG-3′ (forward and reverse primer in the second PCR). The resulting two fragments were used in a further PCR to obtain a complete mutated ROS-GC1-DNA construct (3320 bp) by employing the primers 5′-AAAGCTAGCCACCATGACCGCCTG-CGCCCGCC-3′ and 5′-TCAAGAGAACTGGCCCGGCCGC-3′. The final construct was cut with Nhe I, phosphorylated with T4 polynucleotide kinase and ligated in the pIRES2-EGFP vector that was cut by Nhe I/Sma I. Clone products were verified by sequencing.

### HETEROLOGOUS EXPRESSION OF ROS-GC1 AND GCAPs

WT and mutant forms of ROS-GC1 were expressed in HEK flip 293 cells. For this purpose cells were cultivated in minimal essential medium under standard conditions as described (Koch and Helten, 2008). After culturing 3–5 × 10^6^ cells were harvested in 100 μL of a buffer containing 5 mM KCl, 20 mM MgCl_2_, and 150 mM phosphate buffer pH 7.2. For transfection, 5 μg of DNA was added and cells were electroporated with the CLB system (Lonza). Further treatment of cells and harvesting for subsequent guanylate cyclase assays was done exactly as described (Zägel et al., 2013). GCAP1 and GCAP2 were expressed in *E. coli* and purified to homogeneity by anion exchange and size exclusion chromatography as previously reported (Hwang et al., 2003; Koch and Helten, 2008). Myristoylation of GCAPs during bacterial expression was accomplished by co-transforming *E. coli* cells with *N*-myristoyl-transferase from yeast and supplementation with myristic acid. To ensure a high degree of myristoylation of GCAP1, which lacks the consensus site for yeast *N*-myristoyl-transferase, we used the D^6^S-mutant of GCAP1 (Krylov et al., 1999; Hwang et al., 2003). The degree of myristoylation of both GCAPs was typically higher than 90% as checked by using an analytical HPLC run on a reverse-phase column.

### GUANYLATE CYCLASE ASSAY

Recombinant human ROS-GC1 in HEK cell membranes was measured as described before (Koch and Helten, 2008; Zägel et al., 2013). In addition to measuring the basal guanylate cyclase activity we mainly tested two effects of GCAPs on the activation profile of the cyclase. In a first set we added purified bovine GCAP1 or GCAP2 at saturating 10 μM to washed HEK cell membranes and incubated samples at different free [Ca^2^^+^] using a Ca^2^^+^-EGTA buffer system. Details of the procedure have been described in several previous publications (Hwang et al., 2003; Koch and Helten, 2008; Zägel et al., 2013). Data were evaluated to obtain the [Ca^2^^+^], at which the activation is half-maximal (IC_50_). In a second set of guanylate cyclase assays we incubated recombinant ROS-GC1 with increasing concentrations of GCAP1 and GCAP2 (0–20 μM) for 30 min under Ca^2^^+^-free conditions. GCAPs were liganded with Mg^2^^+^ (Peshenko and Dizhoor, 2004) using the same buffer composition as described (Koch and Helten, 2008). The latter activity measurements yielded apparent affinities (EC_50_-values) of GCAPs for WT and mutant ROS-GC1. Activity values were obtained from at least 3–4 different data sets and were used to calculate the mean ± standard deviation (SD). A student’s *t*-test was performed to check for significant differences between two sets of data.

### IMMUNOHISTOCHEMISTRY

Localization of ROS-GC1 WT and mutants in HEK cells by immunofluorescence microscopy was done as described before using an Olympus fluorescence microscope (Zägel et al., 2013). The following primary antibodies were used for detection: anti-ROS-GC1 (1:100; ROS-GC1 (H-225), sc50512, rabbit polyclonal IgG; Santa Cruz Biotechnology), anti-Na^+^/K^+^-ATPase (1:200; Na^+^/K^+^-ATPase, a (H-3), sc-48345, mouse monoclonal IgG_2b_; Santa Cruz Biotechnology), and anti-calnexin [1:200; calnexin (E-10), sc-46669, mouse monoclonal IgG_2a_; Santa Cruz Biotechnology]. Secondary antibodies were donkey anti-rabbit conjugated to Fura350 (dilution 1:200) from Invitrogen and goat anti-mouse conjugated with Dylight594 from Thermo Scientific, USA used in a dilution of 1:500. Incubation and washing buffers were exactly as described (Zägel et al., 2013).

## RESULTS AND DISCUSSION

### LOCALIZATION OF ROS-GC1 CONSTRUCTS

**FIGURE 2:**
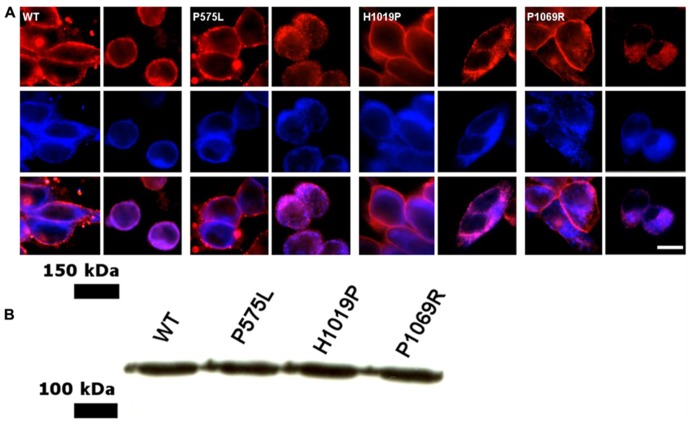
**Cellular localization of WT and ROS-GC1 mutants in HEK293 cells**. **(A)** Cells were stably transfected with human ROS-GC1 constructs and were probed with different antibodies; the corresponding ROS-GC1 construct used for transfection is indicated in the corner of the upper left panel. Anti-Na^+^/K^+^-ATPase was used as plasma membrane marker (upper left panel, red staining); anti-calnexin antibodies as marker for the endoplasmic reticulum (top right panel, red staining); anti-ROS-GC1 antibody (middle panels, blue staining); overlay of ROS-GC1 staining and membrane specific localization (lower panels). Secondary antibodies were indicated in the Methods section. Visualization was done in an Olympus fluorescence microscope. Cells were fixed either with paraformaldehyde (localization of plasma membrane) or with paraformaldehyde-methanol (endoplasmic reticulum). Scale bar = 10 μm. **(B)** Western blot of HEK293 cells expressing human ROS-GC1, WT and the mutants P575L, H1019P, and P1069R. Blots were probed with a primary antibody against ROS-GC1 (GC1#3, dilution 1:2000) and a secondary anti-rabbit antibody from Dianova (dilution 1:4000) as described (Zägel et al., 2013).

Previous work has shown that ROS-GC1 can be expressed heterologously in HEK293 cells in sufficient quantity to allow subsequent biochemical studies (Hwang et al., 2003; Koch and Helten, 2008; Zägel et al., 2013). In HEK293 cells the enzyme was found to be present in cell membranes and mainly co-localized with the endoplasmic reticulum. Probing the cells with an anti-ROS-GC1 antibody (**Figure 2A**, middle panel) and using specific markers for the plasma membrane (anti-Na^+^/K^+^-ATPase antibody, top left panel in **Figure 2A**) and for the endoplasmic reticulum (anti-calnexin, top right panel) we obtained a staining pattern in the overlay image (**Figure 2A**, bottom panels) in agreement with published results (Peshenko et al., 2008; Zägel et al., 2013). The localization of all investigated mutants was very similar to the cellular localization of the WT. Using western blotting we further estimated that the amount of ROS-GC1 WT and mutants was nearly the same in a suspension of cells having a similar cell density (**Figure 2B**, also Zägel et al., 2013).

### BASAL GUANYLATE CYCLASE ACTIVITIES

**FIGURE 3:**
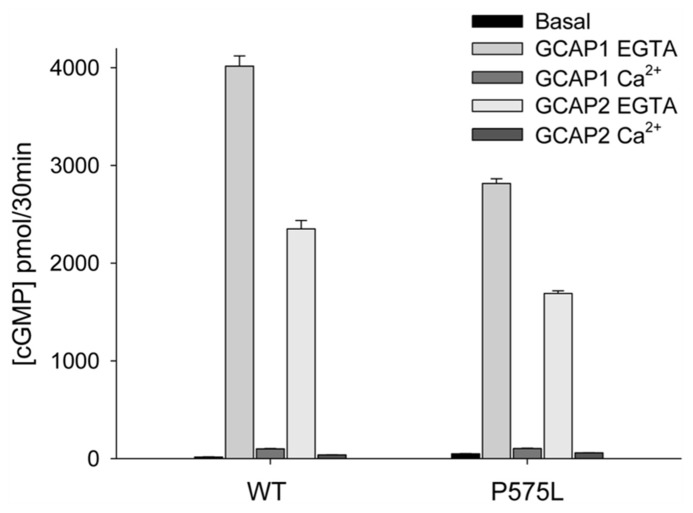
** Guanylate cyclase activities of WT and the P575L mutant**. Ca^2^^+^-bound and Ca^2^^+^-free (EGTA) GCAP1 or GCAP2 were added to HEK293 cell membranes expressing human ROS-GC1 (inset) yielding a final concentration of 10 μM. Ca^2^^+^ was either at 33 μM or <10 nM (EGTA). Assay incubations were performed with equal amounts of WT and mutant guanylate cyclase in membranes, which was verified by western blotting (see **Figure 2B**). Some error bars are not visible due to the scaling of the figure. Differences between data sets were highly significant yielding ***P ≤ 0.001, except for P575L; when its basal activity was compared with the activity in the presence of Ca^2^^+^-bound GCAP2, it resulted in**P ≤ 0.01.

The mutant P575L exhibited similar basal GC activities as WT (**Figure 3**). Activities in the presence of GCAP1 and 2 at saturating [Ca^2^^+^] revealed almost no difference between P575L and WT. However, a difference was observed, when GCAP1 and 2 were incubated with ROS-GC1 at low [Ca^2^^+^] (EGTA columns in **Figure 3**) resulting in a 25–30% decrease of maximal activity. In sharp contrast to P575L, the mutant P1069R did not exhibit any activity, neither in the presence nor in the absence of Ca^2^^+^, EGTA, or GCAPs (data not shown). We also tested activities of cells transiently expressing the mutant or a stably transfected cell line. Since the cellular localization of this mutant appeared normal and was very similar to the active WT and P575L forms (**Figure 2A**), we conclude that the lack of GC activity was a consequence of an impaired catalytic domain.

A special case was observed with the mutant H1019P: we were unable to obtain consistent activity profiles as we did with WT and the P575L mutant. In some cell batches we observed activation by GCAP1, but not by GCAP2, whereas in other batches both GCAPs were able to activate the cyclase or the enzyme was inactive under all conditions. The large scattering of the results seems to indicate a general instability of the mutant.

### GCAP-MEDIATED ACTIVATION

**FIGURE 4:**
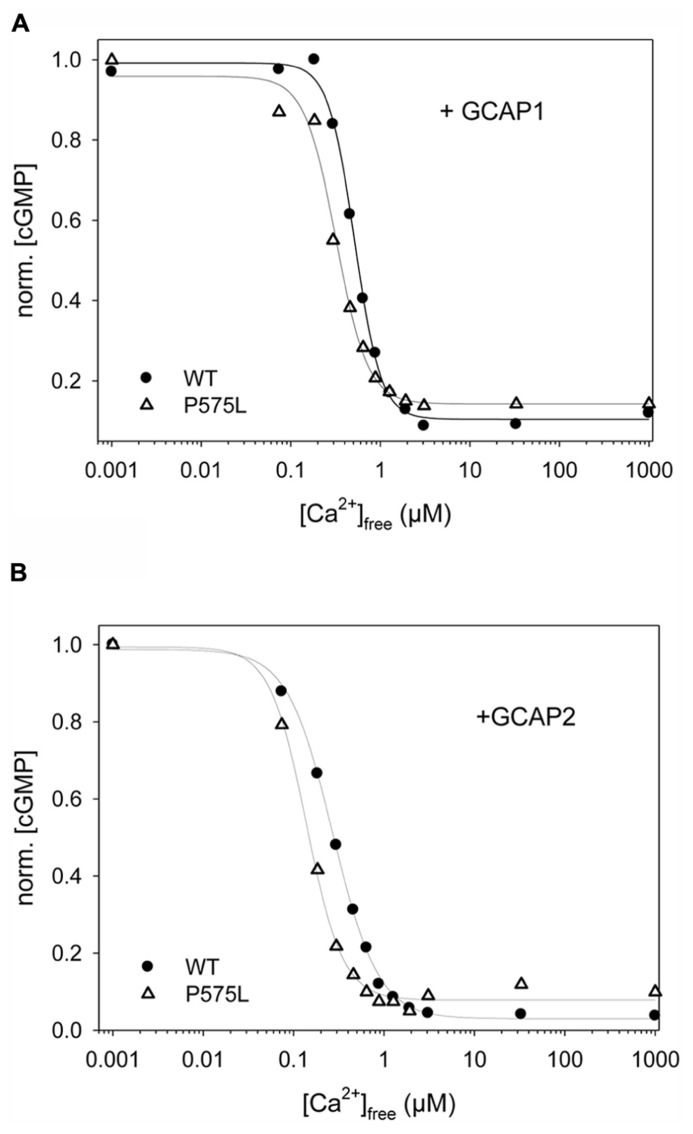
**Ca^2^^+^-sensitive activity profiles of WT and ROS-GC1 mutant P575L**. Guanylate cyclase activities were measured as a function of free [Ca^2^^+^] for samples as indicated **(A)** WT or P575L, in the presence of 10 μM GCAP1. **(B)** WT or P575L, in the presence of 10 μM GCAP2. Incubation time was 30 min. Activities are normalized to allow comparison of IC_50_ values ([Ca^2^^+^]_free_ at which activity is halfmaximal). Data are representative of three to four sets of incubations.

The key feature among ROS-GC1 properties is the Ca^2^^+^-dependent activation under control of GCAPs. We tested this activation of P575L by GCAPs and compared it with the WT activity profile. Representative data sets are shown in **Figure 4A** (+GCAP1) and **Figure 4B** (+GCAP2), a summary of the IC_50_ values (mean ± SD) is displayed in **Table 1**. For both Ca^2^^+^-sensor proteins we observed a similar two-fold shift of the IC_50_ values to lower [Ca^2^^+^], in the presence of GCAP1 from 0.529 (WT) to 0.270 μM (P575L) and in the presence of GCAP2 from 0.269 μ(WT) to 0.128 μM (P575L).

**Table 1:** Summary of IC_50_ and EC_50_ values for WT and P575L mutant.

ROS-GC1	IC_50_ (μM)	EC_50_ (μM)
WT + GCAP1	0.529 ± 0.006	2.93 ± 0.37
WT + GCAP2	0.269 ± 0.009	8.34^+^ ± 0.36
P575L + GCAP1	0.270* ± 0.018	3.32^+^ ± 0.39
P575L + GCAP2	0.128 ± 0.014	7.44 ± 1.26

*Six data sets; ^+^two data sets.

*Values are the mean ± SD of mainly three to four independent measurements, if not indicated otherwise.*

**FIGURE 5:**
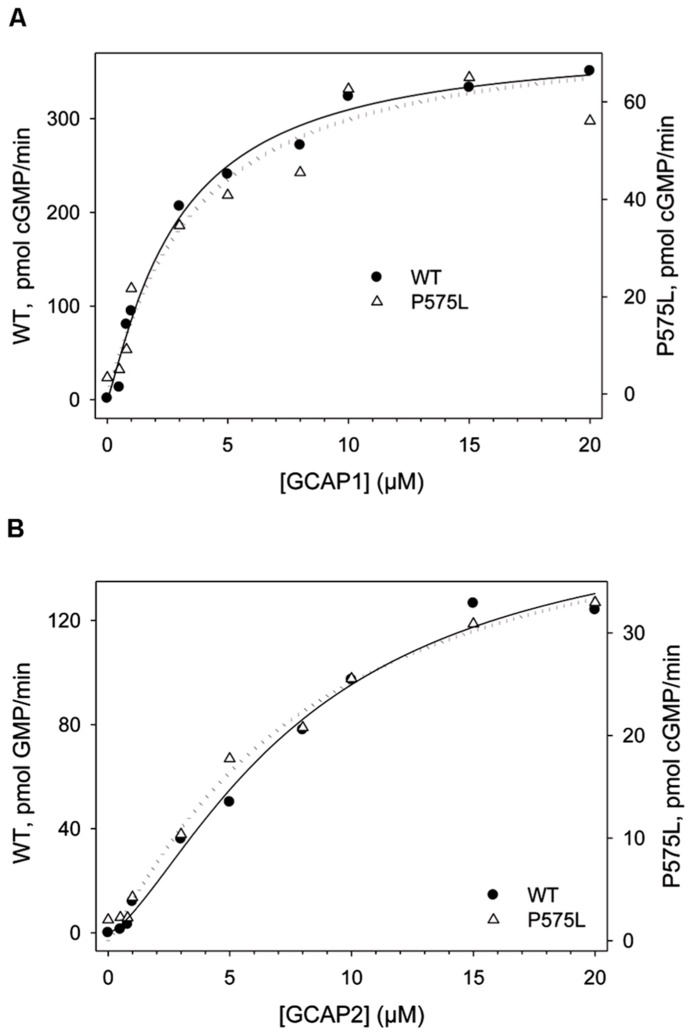
**GCAP-dependency of WT and P575L to determine apparent affinities (EC_50_)**. WT and mutant P575L (see inset) were incubated with Ca^2^^+^-free (Mg^2^^+^-bound) GCAP1 **(A)** and GCAP2 **(B)**. The amount of cGMP produced by ROS-GC1 is shown as a function of the GCAP concentration. Incubation time was 30 min.

A shift in the IC_50_ values had previously been reported for some autosomal cone dystrophy related mutations that are located in a so-called “mutation hotspot” region within or near the residue R838 in the dimerization domain (Duda et al. 1999a, 2000; Wilkie et al., 2000; Kitiratschky et al., 2008; Zägel et al., 2013). One reasonable model explaining this observed effect states that the shift in Ca^2^^+^-sensitivity originates from different apparent affinities of WT and mutant ROS-GC1 for the Ca^2^^+^-free form of GCAPs (Peshenko et al., 2004). We tested this hypothesis for the P575L mutant by measuring the GC activities at increasing GCAP concentrations (**Figure 5**). GCAP1 and 2 were Ca^2^^+^-free, but they had Mg^2^^+^-bound (Peshenko and Dizhoor, 2004) under the assay conditions. Apparent affinities expressed as EC_50_-values differed only minimally between WT and the P575L mutant and overlapped within the measured standard deviations (Table 1). Thus, we conclude that differences in apparent affinities for GCAPs cannot account for the observed shifts in Ca^2^^+^-sensitivity of the P575L mutant.

### STRUCTURE-FUNCTION RELATIONSHIPS

The single point mutation P575L is located in direct C-terminal neighborhood of a small stretch of 20 amino acids, which was previously identified as an essential regulatory and/ or interaction site in bovine ROS-GC1 for GCAP1 (L559–I578 corresponding to peptide #34a in Lange et al., 1999). The region is highly conserved among different species and encompasses also the point mutation F565S (human sequence) that is linked to LCA type 1 (Perrault et al., 1996). However, the biochemical consequences of these two mutations differ significantly, since F565S leads to a more than 10-fold lower basal cyclase synthesis rate, but also to a complete loss of the GCAP1-stimulated GC activity (Duda et al., 1999b). These biochemical consequences are in contrast to the less severe effects caused by the P575L mutation.

The position H1019P is located in the short β6-sheet of the CCD in close vicinity of the β5-sheet that harbors amino acids R995, C997, L998, and F999, which are critical for binding the purine ring of the GTP substrate (Tucker et al., 1998). Replacement of H by P might cause a “kink” of the β6-sheet and therefore disrupts or destabilize the catalytic center. A mutation of A to P in α-synuclein for example was reported to have long-range and short-range effects on the three-dimensional structure including a decrease of β-sheet content (Wise-Scira et al., 2013). Furthermore, the position H1019P is also located in the GCAP2 binding site that was previously narrowed down to the amino acid stretch Y1012-N1037 in the bovine sequence (Duda et al., 2005; Pettelkau et al., 2012). In summary, these results might explain the large scattering of GC activities observed in the *in vitro* assays.

In our test system the mutation P1069R had the most dramatic consequences leading to a completely inactive enzyme. This position is sandwiched between the binding sites of GCAP2 and S100β (Duda et al., 2002) and we can only speculate that it might have the function to integrate diverse signals. Furthermore, it seems to be indispensable for the basal catalysis of ROS-GC1.

## CONCLUSION

Inherited retinal disorders can affect visual function in different manners. In the present study we investigated the biochemical consequences of three point mutations in the GUCY2D gene, which cause different forms of visual impairment. The mutation P575L causes a progressive form of cone degeneration in affected patients, which very often correlates with symptoms of photophobia, decrease of central vision and impairment of color vision. Since the mutation did not lead to a complete loss of guanylate cyclase function, cone cells expressing the mutated ROS-GC1 protein very likely maintain key features of photoreceptor physiology. But since the mutation induces a dysregulation of the Ca^2^^+^-sensitive cyclase activation profile, it seems to have long-term effects in the distortion of the Ca^2^^+^-cGMP homeostasis triggering apoptotic pathways. Similar consequences have been discussed for GCAP1 mutations correlating with certain forms of cone-rod dystrophies (Behnen et al., 2010; Hunt et al., 2010). The other two point mutations that were investigated in the present study correlate with LCA and a juvenile form of retinitis pigmentosa. Both mutations have more severe pathological consequences. Typical clinical features are for example an early onset of the disease in childhood and a strongly reduced or even non-recordable electroretinogram (Perrault et al., 2000; Booji et al., 2005). Clinical manifestations of the point mutations appear to be mirrored in the biochemical properties of the expressed ROS-GC1 protein as there are: synthesis of cGMP by ROS-GC1 is either severely disrupted due to protein instability (H1019P) or completely abolished (P1069R). Thus, light-triggered hydrolysis of cGMP cannot adequately be balanced by the second photoreceptor specific guanylate cyclase (ROS-GC2, GC-F), which has a much lower expression level, at least in cone cells (Olshevskaya et al., 2002; Baehr et al., 2007). The cGMP level in photoreceptor cells would be lower than in non-affected cell. This in turn would keep a lower number of cyclic nucleotide-gated channels open in the dark state. Since the cytoplasmic cGMP pool that is hydrolyzed after illumination is already reduced in the dark, amplitude and kinetics of the photoresponse are expected to be smaller and faster.

## Conflict of Interest Statement

The authors declare that the research was conducted in the absence of any commercial or financial relationships that could be construed as a potential conflict of interest.
